# Association of social isolation and cognitive performance: a longitudinal study using a four-wave nationwide survey

**DOI:** 10.1186/s12889-023-16274-7

**Published:** 2023-07-22

**Authors:** Yanran Duan, Shuai Jiang, Zhao Yin, Sufan Wang, Jinghong Gao, Mengyu Yang, Changying Chen, Hang Fu, Chengzeng Wang

**Affiliations:** 1 The First Affiliated Hospital of Zhengzhou University, 450000, 1 Jianshe Road, Henan Province Zhengzhou, China; 2 Institute for Hospital Management of Henan Province, Zhengzhou, China; 3 Zhengzhou University School of Public Health, Zhengzhou, China

**Keywords:** Longitudinal association, Bivariate latent growth model, Trajectories of changes, Bidirectional, Social isolation, Cognitive performance

## Abstract

**Background:**

This study aimed to examine the bidirectional relationship between social isolation and cognitive performance among Chinese middle-aged and older adults.

**Methods:**

We used four waves of data from the China Health and Retirement Longitudinal Study. A latent growth model (LGM) was applied to examine the association between social isolation and cognitive performance across different characteristics.

**Results:**

In the analysis, we ultimately included 9,367 participants after excluding respondents with missing key variables. Social isolation and cognitive performance showed significant differences across time. After adjusting for the confounders, there was a significant association between higher social isolation and poor cognitive performance (*β* = −1.38, *p* < 0.001), and higher levels of social isolation resulted in a more pronounced decline in cognition over time (*β* = 0.17, *p* < 0.001). Additionally, the path coefficient between the initial level of cognition at baseline and the slope of social isolation was − 0.07 (*p* < 0.001) and 0.01 (*p* = 0.021), respectively. For the correlation between slopes, our study found that females’ cognition scores were more susceptible to social isolation (*β* = − 2.78, *p* < 0.001). Similarly, regarding cognition scores, the influence of social isolation was greater among people with education below the primary level (*β* = − 2.89, *p* = 0.002) or a greater number of chronic diseases (*β* = − 2.56, *p* = 0.001).

**Conclusion:**

Our findings support the bidirectional association between social isolation and cognition. Specifically, higher baseline social isolation and its rate of increase over time contribute to an intensification of cognitive decline at follow-up. Besides, poorer cognitive performance predicted higher social isolation.

**Supplementary Information:**

The online version contains supplementary material available at 10.1186/s12889-023-16274-7.

## Introduction

Alzheimer’s Disease International (ADI) estimates that 75% of dementia patients worldwide are not diagnosed, and in some low-income and middle-income countries, this proportion is as high as 90%. Also by 2050, the number of people diagnosed with dementia will increase to approximately 154 million [1]. China is the world’s most populous country, with a rapid growth of the older populations, the prevalence and disease burden of cognitive impairment will increase. The decline of cognitive performance is one of the earliest and obvious symptoms of Alzheimer’s disease [2, 3], and mild cognitive impairment can result with the possibility of progressing to dementia [4], which all highlights the urgency of studying and preventing cognitive impairment in older Chinese adults.

The effects of relation to later-life cognitive decline of social isolation have been extensively examined [5, 6]. Social isolation refers to the objective aspects of isolation, such as having a minimal number of social contacts, living alone, lacking engagement with others and the wider community or having little involvement in social activities [7]. A recent meta-analysis study found relationship between larger social network size, higher social engagement, and better cognitive functioning [8]. Other researchers have investigated whether poor social relationships are associated with cognitive decline [9, 10]. Furthermore, several comprehensive systematic literature reviews concluded that a lack of social engagement and social contact were associated with incident dementia in the general population [11, 12].

In contrast, one study found that worse baseline cognitive functioning and its rate of decline over time seemed to contribute to an intensification of loneliness at follow-up [13]. It is of great significance to explore the bidirectional connection between social isolation and cognitive change; for example, a decline in cognition over time could be related to an increase (or more increase) in social isolation in the same period. Therefore, longitudinal analysis is consequently attracting more attention to define the dynamic linkage between the two variables over time. The present study evaluated possible variations in the relationship between social isolation and cognitive performance in a sample of middle-aged and older adults followed for up 8 years.

Generally, in the relationship between social isolation and cognitive performance, gender is typically ignored as a possible moderator and only included as a covariate [14]. Previous studies have shown that males tend to appear together with more people than females, forming large and coherent group [15], which may optimize cognitive function through access to novel and diverse social stimuli, including a range of ideas, information, activities, verbal and nonverbal social cues, faces, and speech patterns [16]. Therefore, it is worthwhile to investigate whether there are gender differences in the relationship between social isolation and cognitive performance. Moreover, other characteristics may also affect the relationship between them, we stratified the analyses by gender, education level, and health burden in this study.

## Methods

### Study population

In our study, the data were from the China Health and Retirement Longitudinal Study (CHARLS). The baseline national wave of CHARLS was administered in 2011, and the individuals were followed up every two years (1st wave in 2011, 2nd wave in 2013, 3rd wave in 2015, and 4th wave in 2018). The survey aimed to collect data on the family life and community conditions to analyze population problems for people aged 45 and above, especially those related to population aging. Description of the CHARLS and details of the sampling procedure are available elsewhere [17].

**Fig. 1:**
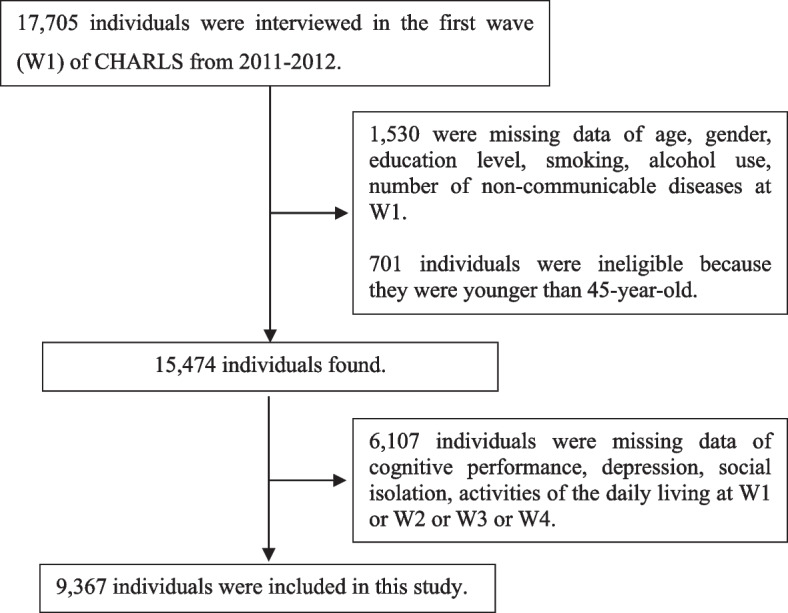
Participants’ flow in the study

From 2011 to 2012, there were 17,705 participants in the national baseline survey. Among the study participants, we excluded participants without information on important exposure-related variables at baseline (*n* = 1,530), and 701 participants were ineligible because they were younger than 45 years old. Next, 6,107 participants had missing data on cognitive performance, depression, social isolation, activities of daily living in 2011 and follow-ups. Ultimately, a nationally representative sample of 9,367 participants were included in the final analysis. The participant flow in the study is shown in Fig. 1.

### Measures in CHARLS

#### Cognitive performance

The Mini-Mental State Examination (MMSE) and more complex measures of mild cognitive impairment can be used as predictors of dementia risk, within a population-based setting [18]. As an adapted version of the MMSE, the Telephone Interview for Cognitive Status (TICS) has been deployed as part of the Health and Retirement Study (HRS) [19]. The CHARLS includes items that assess cognitive function similar to those used in the HRS. Meanwhile, we relied on three composite measures of cognitive functioning, as reported in a previous study [20] (detailed in Supplementary eMethod 1). The scale included (1) TICS: the score of this dimension was calculated according to the number of correct answers, from 0 to 10; (2) word recall: the word recall score was based on the average number of correct answers, from 0 to 10; (3) successfully draw a graph: the respondents who successfully draw a graph would get 1 point, and the respondents who fail to draw a graph would get 0 point. This was a comprehensive measure of respondents’ cognitive performance. We used the sum of the above three indicators to represent the overall cognitive state of respondents, ranging from 0 to 21. Higher scores demonstrate better cognitive performance.

#### Social isolation

We have created an index of social isolation by three items, which was adapted from previous research [21, 22] (detailed in Supplementary eMethod 2). The scale included (1) whether the participants lived together; (2) how often the participants or their spouses saw a parents or in-laws; (3) how often the participant saw or touched his or her child; (4) whether the respondent had interacted with friends in the last month; and (5) whether the participants had participated in any activities in the last month. Scores ranged from 0 to 5, with higher scores indicating higher degree of social isolation.

#### Covariates

The confounding factors in this study included demographic characteristics, lifestyle factors, depression, and activities of daily living (ADL). We examined possible confounding factors through time constants and time-varying covariates of baseline (2011) and initial follow-ups (2013, 2015 and 2018).

We used five types of instrumental activities of daily living (IADL) and six types of ADL to create the daily living activity index. The ten-item Center for Epidemiologic Studies Depression Scale short forms, considered to be an effective and reliable tool for evaluating depression in China, were used to evaluate the depression symptoms of the respondents [23]. Demographic characteristics included age, gender and educational level. Lifestyle behaviors included smoking and alcohol use. We also included health burden based on the number of chronic diseases (NCDs) diagnosed by clinicians. In total, fourteen types of chronic disease conditions were considered in CHARLS, including hypertension, dyslipidemia, diabetes, cancer and so on. The definitions of variables are summarized in Supplementary eTable 1.

### Data analysis

#### Descriptive statistics

Descriptive statistics were presented as the mean (standard deviation of the mean) for continuous variables. Using Spearman’s correlation analysis determined the correlations between social isolation scores and cognitive performance at four waves. One-way repeated measures analysis of variance was applied to assess the changes in the frequency of social isolation scores and cognitive performance in 2011, 2013, 2015, and 2018 for different gender, education level, and health burden groups.

#### Latent growth model

A latent growth model (LGM) was used to examine the trajectories of changes in social isolation scores and cognitive performance. For example, the latent intercept growth factor and the latent slope growth factor reflected the trajectory of change in cognition across time which represented the initial status and rate of change in cognition, respectively [24].

**Fig. 2:**
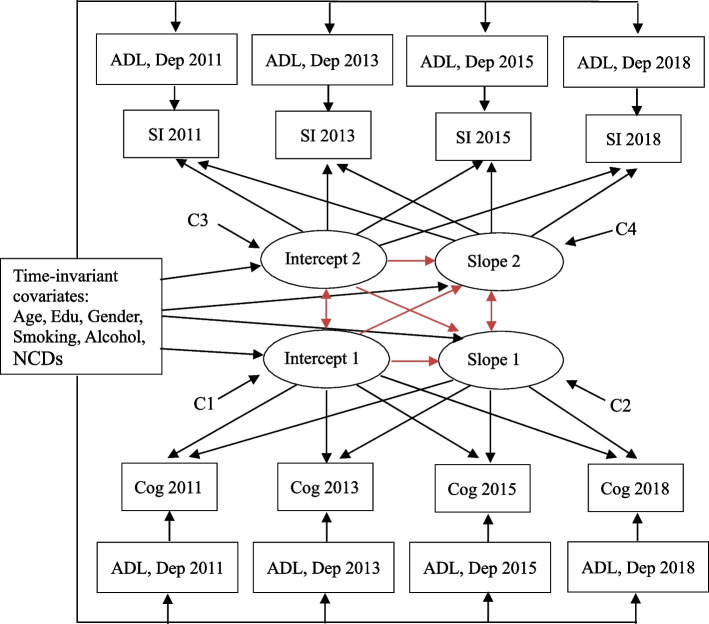
Bivariate latent growth model to assess the relationships of the changes in social isolation and cognition. Cog Cognition, SI Social isolation, ADL Activities of the daily living, Dep Depression, C1-C2 Correlation of the intercept 1 and slope 1. Edu Education level. NCDs number of non-communicable diseases

A univariate latent growth model was tested for both cognitive performance and social isolation scores. As a linear model, time was coded as 0, 1, 2, and 3 for each of the four waves, with wave 1 as the intercept. Next, a bivariate growth curve (Fig. 2) was used for social isolation scores and cognitive performance. We considered controlling for age, gender, education level, smoking, alcohol, and the number of chronic diseases as time invariant covariates at wave 1. ADL and depression were entered as time-variant covariates at each wave.

The bivariate growth curve contained 6 effects of interest: the correlation between intercepts (or wave 1 cognition and social isolation); the correlation between the social isolation intercept and cognition slope, which determines whether social isolation was a risk factor for declining cognition; the correlation between the cognition intercept and social isolation slope, which tests whether poor cognition was a risk factor for increased social isolation; and the correlation between slopes.

The following indices were used to assess the goodness of model fit: chi-square statistic, comparative fit index (CFI) ≥ 0.95, tucker‒lewis index (TLI) ≥ 0.95, standardized root mean square residual (SRMR) ≤ 0.50, and root mean square error of approximation (RMSEA) ≤ 0.08, with 90% CI ≤ 0.08 [25]. Analyses were conducted using Mplus 7.1 (Munthen & Munthen, Los Angeles, CA).

## Results

### Descriptive analysis

Table 1 presents the correlations of cognition with social isolation scores at 4 waves in 2011–2018. Higher social isolation scores were significantly associated with lower of cognitive performance. Further analyses were conducted to examine this negative correlation between social isolation and cognition in this longitudinal study.

**Table 1:** Correlations of cognitive performance with social isolation in Chinese elderly persons at each time point during 2011–2018

	Cog2011	Cog2013	Cog2015	Cog2018	SI2011	SI2013	SI2015	SI2018
Cog2011	1.00							
Cog2013	0.62 ^*a*^	1.00						
Cog2015	0.63 ^*a*^	0.69 ^*a*^	1.00					
Cog2018	0.60 ^*a*^	0.66 ^*a*^	0.69 ^*a*^	1.00				
SI2011	-0.23 ^*a*^	-0.20 ^*a*^	-0.20 ^*a*^	-0.19 ^*a*^	1.00			
SI2013	-0.22 ^*a*^	-0.24 ^*a*^	-0.22 ^*a*^	-0.21 ^*a*^	0.32 ^*a*^	1.00		
SI2015	-0.21 ^*a*^	-0.21 ^*a*^	-0.24 ^*a*^	-0.22 ^*a*^	0.33 ^*a*^	0.37 ^*a*^	1.00	
SI2018	-0.21 ^*a*^	-0.22 ^*a*^	-0.23 ^*a*^	-0.25 ^*a*^	0.29 ^*a*^	0.34 ^*a*^	0.38 ^*a*^	1.00

*Abbreviations*: *Cog *Cognition, *SI *Social isolation

^*a*^*p* < 0.01

Changes in cognition and social isolation were summarized for the total sample based on age, gender, education level, and health burden group at 4 time points, as shown in Table 2. During this study, the cognitive performance of older participants showed a general decreasing trend from 2011 to 2018, with an opposite trend in social isolation scores. One-way repeated measurement analysis showed that cognition changed significantly over time. The cognitive performance and social isolation among older individuals showed a sharp decline and increase related to age, respectively.

**Table 2:** Levels of cognitive performance and social isolation in elderly Chinese participants at each time point

Variable	No. of participants	2011	2013	2015	2018
**Cognition score**	*F* = 1662.07, *p* < 0.001^*a*^				
Total	9367	10.99$\pm$4.21	11.08$\pm$4.21	10.64$\pm$4.35	8.97$\pm$4.36
Age, years					
45 ~ 59	5478	11.53$\pm$4.04	11.66$\pm$4.00	11.36$\pm$4.08	9.34$\pm$4.18
60 ~ 64	1833	10.75$\pm$4.24	10.90$\pm$4.14	10.42$\pm$4.29	8.76$\pm$4.35
65 ~ 79	1980	9.80$\pm$4.35	9.76$\pm$4.46	8.98$\pm$4.56	7.46$\pm$4.48
≥ 80	76	8.34$\pm$4.55	7.99$\pm$4.74	6.79$\pm$4.73	5.14$\pm$4.25
Gender					
Male	4341	12.05$\pm$3.75	12.15$\pm$3.7	11.66$\pm$3.82	9.79$\pm$3.98
Female	5026	10.08$\pm$4.38	10.15$\pm$4.4	9.76$\pm$4.57	7.94$\pm$4.49
Education level ^*b*^					
Level 1	4218	8.68$\pm$3.98	8.70$\pm$3.99	8.07$\pm$4.11	6.20$\pm$3.83
Level 2	4802	12.74$\pm$3.38	12.88$\pm$3.27	12.59$\pm$3.27	10.77$\pm$3.53
Level 3	347	14.83$\pm$2.69	15.13$\pm$2.62	14.80$\pm$2.61	12.97$\pm$2.86
Health burden^*c*^					
None	3023	11.41$\pm$4.20	11.35$\pm$4.18	10.90$\pm$4.34	9.02$\pm$4.31
Mild	4663	10.85$\pm$4.20	11.00$\pm$4.23	10.56$\pm$4.35	8.67$\pm$4.38
Severe	1681	10.61$\pm$4.21	10.81$\pm$4.18	10.37$\pm$4.32	8.74$\pm$4.37
**Social isolation score**	*F* = 91.21, *p* < 0.001^*a*^				
Total	9367	2.12$\pm$1.12	2.02$\pm$1.09	2.14$\pm$1.13	2.24$\pm$1.15
Age, years					
45 ~ 59	5478	2.00$\pm$1.09	1.91$\pm$1.06	1.99$\pm$1.11	2.07$\pm$1.11
60 ~ 64	1833	2.19$\pm$1.11	2.06$\pm$1.08	2.24$\pm$1.10	2.33$\pm$1.13
65 ~ 79	1980	2.37$\pm$1.14	2.26$\pm$1.12	2.42$\pm$1.14	2.59$\pm$1.16
≥ 80	76	2.62$\pm$1.21	2.64$\pm$1.25	2.72$\pm$1.23	3.24$\pm$1.07
Gender					
Male	4341	2.07$\pm$1.11	1.96$\pm$1.08	2.07$\pm$1.13	2.19$\pm$1.14
Female	5026	2.17$\pm$1.12	2.08$\pm$1.09	2.20$\pm$1.12	2.29$\pm$1.16
Education level					
Level 1	4218	2.30$\pm$1.11	2.25$\pm$1.09	2.38$\pm$1.11	2.48$\pm$1.14
Level 2	4802	2.01$\pm$1.10	1.87$\pm$1.06	1.96$\pm$1.11	2.08$\pm$1.12
Level 3	347	1.46$\pm$0.95	1.43$\pm$0.80	1.62$\pm$0.98	1.62$\pm$1.01
Health burden					
None	3023	2.11$\pm$1.10	2.00$\pm$1.08	2.10$\pm$1.13	2.21$\pm$1.15
Mild	4663	2.11$\pm$1.12	2.02$\pm$1.09	2.15$\pm$1.13	2.24$\pm$1.15
Severe	1681	2.16$\pm$1.13	2.07$\pm$1.11	2.17$\pm$1.13	2.28$\pm$1.15

^*a*^*p* value was calculated by one-way repeated measures ANOVA.

^*b*^ Education level, Level 1: Below primary level; Level 2: Elementary/Middle/High school; Level 3: Above senior high school level

^*c*^ Health burden, None (no NCD); Mild (1 or 2 types of NCDs); Severe (more than 3 types of NCDs)

Males exhibited better cognitive performance and lower social isolation scores than females across time. More detailed descriptive statistics at baseline by gender were summarized in Supplementary eTable 2. Regarding cognitive performance, males presented a higher mean score than females. In addition, males were more likely than females to drink (nearly five times) and smoke (nearly ten times), and males had a better education with a senior high school level (5.71%) than female (1.97%).

### Univariate growth curves

The measurement results and structural model are summarized in Table 3. Linear LGM was used to describe the change track of cognition and social isolation. The results showed that the intercept and slope were both significant, meaning that the average change rate of cognition from 2011 to 2018 was a typical decreasing trend. In addition, the initial status of LGM was 11.50, which was similar to the cognitive performance in 2011 (10.99). The intercept of the social isolation growth track showed that the initial social isolation score level was 3.22 (*p* < 0.001). The estimated slope was 0.36 (*p* < 0.001) indicating that the change rate of social isolation increased significantly in the four waves.

**Table 3:** Coefficients for measurement models

Models	Parameters	Coefficients	Z value	*p* value	Goodness-of-fit indices
Trajectory of Cog	Intercept	11.50	278.857	< 0.001	$\chi$^2^(5) = 1472.05, *p* < 0.001; CFI = 0.91, TLI = 0.89, SRMR = 0.046, RMSEA = 0.177 (0.169, 0.185)
	Slope	-0.72	-54.449	< 0.001	
Trajectory of SI	Intercept	3.22	47.426	< 0.001	$\chi$^2^(5) = 162.19, *p* < 0.001; CFI = 0.97, TLI = 0.96, SRMR = 0.031, RMSEA = 0.058 (0.050, 0.066)
	Slope	0.36	6.890	< 0.001	

*Abbreviations*: *Cog *Cognition, *SI *Social isolation, *CFI *Comparative fit index, *TLI *Tucker-Lewis index, *SRMR *Standardized root mean square residual, *RMSEA *Root mean square error of approximation

### Bivariate growth curves

Table 4 presents the conditional structural models to assess the two variables that were dynamically linked in some way, and established with satisfactory model fitness. The intercepts of social isolation and cognition were negatively correlated. The path coefficient between the two intercepts was − 1.38 (*p* < 0.001), which indicated the initially higher social isolation and the lower cognitive performance. Change in social isolation over time was negatively associated with the change in cognition (*β* = − 2.27, *p* < 0.001). Therefore, compared with others, participants who had a small increase in social isolation tended to show a greater decline in cognitive performance. The path coefficient of the intercept of social isolation showed a significant association with the rate of change in cognition (*β* = 0.17, *p* < 0.001), suggesting that participants with higher scores on social isolation had a more pronounced decline in cognitive performance over time and vice versa.

**Table 4:** Coefficients for conditional models ^*a*^

	Parameters	Coefficients	Z value	*p* value	Goodness-of-fit indices
Cognition	SI intercept →Cog intercept	-1.38	-17.344	< 0.001	$\chi$^2^(104) = 1227.725, *p* < 0.001; CFI = 0.96, TLI = 0.95, SRMR = 0.030; RMSEA = 0.034 (0.032, 0.036)
	SI intercept →Cog slope	0.17	4.986	< 0.001	
	SI slope →Cog slope	-2.27	-4.835	< 0.001	
Social isolation	Cog intercept →SI intercept	-0.07	-15.436	< 0.001	$\chi$^2^(104) = 2835.938, *p* < 0.001; CFI = 0.91, TLI = 0.88, SRMR = 0.039, RMSEA = 0.053 (0.051, 0.055)
	Cog intercept →SI slope	0.01	2.312	0.021	
	Cog slope →SI slope	-0.20	-3.801	< 0.001	

*Abbreviations*: *Cog *Cognition, *SI *Social isolation, *CFI *Comparative fit index, *TLI *Tucker-Lewis index, *SRMR *Standardized root mean square residual, *RMSEA *Root mean square error of approximation

^*a*^ Adjusted for age, gender, education level, smoking, alcohol use, number of non-communicable diseases, activities of daily living, and depression symptoms

Additionally, the path coefficient between the initial level of cognition at baseline and the slope of social isolation was − 0.07 (*p* < 0.001) and 0.01 (*p* = 0.021), respectively. The slope of cognition and the slope of social isolation were significantly (negatively) correlated, which means that participants who had better cognition than others tended to show more stable social isolation scores (*β* = − 0.20, *p* < 0.001).

### Subgroup analysis

The directional association between social isolation and cognitive performance for each gender group was similar to the entire sample in Supplementary eTables 3–5. Compared with males (*β* = − 1.43, *p* = 0.015), females’ cognition scores were more susceptible to social isolation (*β* = − 2.78, *p* < 0.001). Similarly, regarding cognition score, those people with education below the primary level or a greater number of chronic diseases were more susceptible to social isolation (*β* = − 2.89, *p* = 0.002; *β* = − 2.56, *p* = 0.001), respectively.

## Discussion

Our study aimed to explore the longitudinal relationship between social isolation and cognitive performance and pay attention to examine the covariable of change in the stage of older adulthood, such as activities of daily living and depression. On the whole, the path effects confirmed that the association between social isolation and cognition was bidirectional. Additionally, gender-, education-, and diseases-based differences in trajectories were reported. Interventions of social prescribing, an integrated, multifaceted, and concerted approach, could help to alleviate the problems of social isolation, cognitive impairment and its manifestations.

### Cognitive performance and social isolation interrelate

In our study, the results indicated that social isolation is associated with cognitive performance in later life, consistent with previous studies indicating that lower social engagement or social networks lead to a greater risk of cognitive decline in older adults [26]. Enhancing brain processes can create a buffer against cognitive decline through participation in social and cognitively stimulating activities [27]. There have been proposed several theories to explain the association between social isolation and cognitive performance. One theory is cognitive reserve, which argues that when individuals receive more cognitive stimulation through social contact, cognitive reserve enhances and benefits cognitive function [5]. Another theory is stress-buffering, which proposes that stress is associated with cognitive decline due to structural changes in the hippocampus [28], while social relationships may prevent or modulate responses to stressful events, buffering potentially adverse impacts [29].

Our investigations found evidence of the plausibility of both pathways. In our finding, the change in cognitive performance leading to the changes in social isolation is equally as plausible as the more common finding that social isolation results in cognitive decline. Specifically, the cognitive level was associated with changes in social isolation, and significantly, the lower cognitive was associated with a more pronounced increase in social isolation over time. Correspondingly, only a few studies have indicated that cognition may affect objective and perceived social isolation in healthy individuals [30]. Besides, another study reported that people with mild cognitive impairment tend to use avoid social engagement as a coping mechanism and accompany by a lower quality of life, greater symptoms of depression [31].

### Subgroup analysis

Regarding its associations with cognitive development and maintenance, social isolation appears to be more impactful among females than males at the cognitive level, which was consistent with other research [32]. Several theories have been proposed to explain this difference. There is a discrepancy in social roles; males tend to form larger groups than females, thus stimulating intelligence and buffering against cognitive decline [15]. Another possibility was that providing emotional support and keeping the context of close relationships with similar others could buffer the impact of social isolation on cognitive impairment, while men were more likely than females to have confidants, thus contributing to greater emotional support [33].

In addition, our study found that education level appears to play an important role in the association of social isolation and cognition. As one study has reported [34], for individuals with higher levels of education, spending more time reading or engaging in other intellectual pursuits may compensate for the lack of social contact.

Also, one research suggested that working in high mental-demand jobs could offset the adverse association between social isolation and cognitive functioning [35]. Similar, another research indicated that loneliness was associated to cognitive impairment, adjusted by age, gender, education level, number of chronic diseases, and so on [36]. With the growth of age, older adults are facing multiple chronic diseases problems, which have a negative impact on their functional impairment.

### Prevention and intervention

Traditionally, older adults in China are more likely to live with their children and establish contact with their families or relatives guided by the cultural traditions of familism [37], leading to rather limited social activities. Therefore, it is vital that guidance on how to address the health risks associated with social isolation can be added to the education of health care professionals to promote the prevention and treatment of the cognitive impairment among individuals with poor social relations.

Social prescribing draws from and promotes the usage of community resources and provides individuals with the most appropriate care, showing promise of improving social and psychological wellbeing. A social prescription could be participating in an exercise group, joining a bereavement network, taking an art or dance class, exploring a local hiking trail with a group of peers and much more [38]. Therefore, it is very helpful for improving the mental health of people with social isolation and loneliness. Additionally, as for people with cognitive impairment and dementia, being in a dementia choir designed to be sociable as well as a brain-boosting activity is probably one of the best-known examples of social prescribing.

### Strengths and limitations

To the best of our knowledge, this was the first study in China to investigate the potential bidirectional association between social isolation and cognitive performance in middle-aged and older adults. The CHARLS is a large-scale prospective cohort study, which provide a unique opportunity to test our research issues. Social isolation was measured by five items, which had a better predictive validity than the single-item assessment.

There were some limitations in this study. First, the measurement of cognitive function might not be sensitive enough to identify early-stage cognitive impairment, without clinical diagnosis or other cognitive tests. Second, the bivariate LGM did not allow to infer the time sequence between variables. In addition, some participants dropped out after the baseline wave, and thus, the estimates presented herein are more conservative than the true associations.

## Conclusion

Our study found that social isolation was linked with cognitive decline; in contrast, changes in cognitive performance caused to changes in social isolation. These findings expand our knowledge about the bidirectional association of social isolation with cognitive performance. Considering cognitive decline is a strong risk factor for the development of dementia. Reducing isolation through social prescribing may therefore have substantial benefits in terms of preventing dementia for Chinese older adults.

## Supplementary Information

**Additional file 1.**

## Data Availability

The datasets generated and/or analysed during the current study are available in the CHARLS repository, https://charls.charlsdata.com/index/zh-cn.html.
