# Supplementary material for: Association of social isolation and cognitive performance: a longitudinal study using a four-wave nationwide survey
Source: BMC Public Health. 2023 Jul 22;23:1409. doi: 10.1186/s12889-023-16274-7 (PMC10362697; doi:10.1186/s12889-023-16274-7)
Supplement: Supplementary file 1 — Additional file 1. [file 12889_2023_16274_MOESM1_ESM.docx]

**Association of social isolation and cognitive performance: a longitudinal study using a four-wave nationwide survey**

**Table of contents**

eMethod 1: Measures of cognitive performance

eMethod 2: Measures of social isolation

eTable 1. Definitions of variables used

eTable 2. Descriptive statistics of the baseline sample

eTable 3. Coefficients for structural models by gender

eTable 4. Coefficients for structural models by education level

eTable 5. Coefficients for structural models by health burden

## eMethod 1: Measures of cognitive performance

Three composite measures were combined to create an index of cognitive performance:

(1) Telephone Interview for Cognitive Status (TICS): TICS reflects the mental status of cognition and involves ten questions, including recalling today’s date (month, day, year), the day of the week and season of the year, and serial 7 subtraction from 100 (up to five times). This dimension score is calculated on the number of correct answers, ranging from 0 to 10.

(2) Word recall: The second measure of cognition relies on word recall of ten words, mainly testing episodic memory of cognition. After the interviewer reads a list of ten Chinese words, the participant is asked to repeat the words in any order immediately. About 4 minutes later, the respondent is asked to recall the list of words again. The word recall score is based on the average of the number of correct answers, ranging from 0 to 10.

(3) Drawing a figure successfully: The third cognitive measure is a test of the ability to draw a picture of two overlapping pentagons. Respondents who successfully reduce the picture receive a score of 1, and those who fail to do so receive a score of 0. This is an overall measure of the respondent’s cognitive function.

Scores ranged from 0 to 21, with higher scores indicating better cognition.

## eMethod 2: Measures of social isolation

Five items were combined to create an index of social isolation:

(1) Whether the participant was cohabiting or not, measured using responses to questions on marital status and “living with a partner,” with 1 point assigned to persons answering that they were not married and not cohabiting.

(2) How often the participant or his/her spouse saw a parent or in-law.

(3) How often the participant saw or contacted (including by phone, text message, mail, or e-mail) his/her child.

(4) Whether the participant had interacted with friends in the last month, with 1 point each assigned to respondents who had less than monthly contact with children, parents, or friends.

(5) Whether the participant had engaged in any activities (such as social clubs or resident groups, religious groups, or committees) in the last month, with 1 point assigned to respondents who did not participate in any activities.

Scores ranged from 0 to 5, with higher scores indicating greater social isolation.

## eTable 1. Definitions of variables used

| **Baseline**  **characteristics** | **Wave** | **Definitions** |
| --- | --- | --- |
| Time-invariant | Wave 1  (2011–2012) |  |
| Age |  | Continuous |
| Gender |  | Male = 1, Female = 2 |
| Education level |  | 3 Categories: “Below primary level” “Elementary/Middle/High school”  “Above senior high school level” |
| Smoking *^a^* |  | Yes = 1, No = 2 |
| Alcohol use |  | Drink more than once per month = 1, Drink less than once per month = 2, None of these = 3 |
| Health burden |  | 3 Categories: the number of chronic diseases (NCDs). “None” (no NCD), “Mild” (1 or 2 types of NCDs) or “Severe” (more than three types of NCDs). |
| Time-variant | Waves 1–4  (2011–2018) |  |
| Cognitive performance scores | | Continuous variable ranging from 0 to 21 |
| Activities of daily living (ADL) | | Continuous variable ranging from 0 to 33 |
| Depression scores | | Continuous variable ranging from 0 to 30 |
| Social isolation scores | | Continuous variable ranging from 0 to 5 |

*^a^* Smoking means smoking more than 100 cigarettes in life.

## eTable 2. Descriptive statistics of the baseline sample (N = 9,367)

| **Variable** | **Male** | **Female** | **Total** |
| --- | --- | --- | --- |
| Time-invariant *^a^* |  |  |  |
| Age, years |  |  |  |
| 45~59 | 2418(55.70) | 3060(60.88) | 5478(58.48) |
| 60~64 | 887(20.43) | 946(18.82) | 1833(19.57) |
| 65~79 | 1005(23.15) | 975(19.40) | 1980(21.14) |
| ≥80 | 31(0.71) | 45(0.90) | 76(0.81) |
| Education level |  |  |  |
| Below primary level | 1284(29.58) | 2934(58.38) | 4218(45.03) |
| Elementary/Middle/High school | 2809(64.71) | 1993(39.65) | 4802(51.27) |
| Above senior high school level | 248(5.71) | 99(1.97) | 347(3.70) |
| Smoking |  |  |  |
| Yes | 3210(73.95) | 385(7.66) | 3595(38.38) |
| No | 1131(26.05) | 4641(92.34) | 5772(61.62) |
| Alcohol use |  |  |  |
| Drink more than once per month | 1992(45.89) | 362(7.20) | 2354(25.13) |
| Drink less than once per month | 478(11.01) | 258(5.13) | 736(7.86) |
| None of these | 1871(43.10) | 4406(87.66) | 6277(67.01) |
| Health burden |  |  |  |
| None (no NCD) | 1502(34.60) | 1521(30.26) | 3023(32.27) |
| Mild (1 or 2 types of NCDs) | 2168(49.94) | 2495(49.64) | 4663(49.78) |
| Severe (more than 3 types of NCDs) | 671(15.46) | 1010(20.10) | 1681(17.95) |
| Time-variant *^b^* |  |  |  |
| Cognitive performance scores | 12.05(3.75) | 10.08(4.38) | 10.99(4.21) |
| Activities of daily living (ADL) | 0.78(2.49) | 1.14(2.77) | 0.97(2.65) |
| Depression scores | 8.97(4.44) | 10.73(5.07) | 9.91(4.87) |
| Social isolation scores | 2.07(1.11) | 2.17(1.12) | 2.12(1.12) |

Abbreviations: NCDs number of chronic diseases. *^a^* number (percent). *^b^* mean (standard deviation).

## eTable 3. Coefficients for structural models by gender

| **Conditional model *^a^*** | **Parameters** | **Coefficients** | **Z value** | ***P* value** | **Goodness-of-fit indices** |
| --- | --- | --- | --- | --- | --- |
| Male |  |  |  |  |  |
|  | SI intercept →Cog intercept | -1.29 | -12.352 | < 0.001 | $\chi$^2^(100) = 566.36, *P* < 0.001; CFI = 0.96, TLI = 0.95, SRMR =0.031, RMSEA = 0.033 (0.030, 0.035) |
|  | SI intercept →Cog slope | 0.10 | 2.142 | 0.032 |  |
|  | SI slope →Cog slope | -1.43 | -2.429 | 0.015 |  |
|  |  |  |  |  |  |
|  | Cog intercept →SI intercept | -0.10 | -12.337 | < 0.001 | $\chi$^2^(100) = 1262.99, *P* < 0.001; CFI = 0.90, TLI = 0.87, SRMR =0.042, RMSEA = 0.052 (0.049, 0.054) |
|  | Cog intercept →SI slope | 0.01 | 2.031 | 0.042 |  |
|  | Cog slope →SI slope | -0.12 | -2.190 | 0.029 |  |
| Female |  |  |  |  |  |
|  | SI intercept →Cog intercept | -1.48 | -12.478 | < 0.001 | $\chi$^2^(100) = 747.28, *P* < 0.001; CFI = 0.96, TLI = 0.95, SRMR =0.033, RMSEA = 0.036 (0.034, 0.038) |
|  | SI intercept →Cog slope | 0.23 | 4.592 | < 0.001 |  |
|  | SI slope →Cog slope | -2.78 | -4.013 | < 0.001 |  |
|  |  |  |  |  |  |
|  | Cog intercept →SI intercept | -0.06 | -10.376 | < 0.001 | $\chi$^2^(100) = 1651.55, *P* < 0.001; CFI = 0.91, TLI = 0.89, SRMR =0.042, RMSEA = 0.056 (0.053, 0.058) |
|  | Cog intercept →SI slope | 0.01 | 1.506 | 0.132 |  |
|  | Cog slope →SI slope | -0.31 | -2.596 | 0.009 |  |
| *^a^* Adjusted for age, education level, smoking, alcohol use, number of non-communicable diseases, activities of daily living, and depression symptoms. | | | | | |

## eTable 4. Coefficients for structural models by education level

| **Conditional model *^a^*** | **Parameters** | **Coefficients** | **Z value** | ***P* value** | **Goodness-of-fit indices** |
| --- | --- | --- | --- | --- | --- |
| Below primary level |  |  |  |  |  |
|  | SI intercept →Cog intercept | -1.63 | -12.421 | < 0.001 | $\chi$^2^(100) = 622.64, *P* < 0.001; CFI = 0.95, TLI = 0.93, SRMR =0.034, RMSEA = 0.035 (0.033, 0.038) |
|  | SI intercept →Cog slope | 0.21 | 3.932 | < 0.001 |  |
|  | SI slope →Cog slope | -2.89 | -3.085 | 0.002 |  |
|  | Cog intercept →SI intercept | -0.06 | -10.431 | < 0.001 | $\chi$^2^(100) = 1353.05, *P* < 0.001; CFI = 0.88, TLI = 0.84, SRMR =0.045, RMSEA = 0.055 (0.052, 0.057) |
|  | Cog intercept →SI slope | 0.01 | 1.438 | 0.151 |  |
|  | Cog slope →SI slope | -0.22 | -2.516 | 0.012 |  |
| Elementary/Middle/High school | |  |  |  |  |
|  | SI intercept →Cog intercept | -1.23 | -12.420 | < 0.001 | $\chi$^2^(100) = 644.23, *P* < 0.001; CFI = 0.93, TLI = 0.91, SRMR =0.037, RMSEA = 0.034 (0.031, 0.036) |
|  | SI intercept →Cog slope | 0.13 | 2.815 | 0.005 |  |
|  | SI slope →Cog slope | -1.77 | -6.180 | 0.001 |  |
|  | Cog intercept →SI intercept | -0.10 | -12.288 | < 0.001 | $\chi$^2^(100) = 1517.03, *P* < 0.001; CFI = 0.82, TLI = 0.76, SRMR =0.053, RMSEA = 0.054 (0.052, 0.057) |
|  | Cog intercept →SI slope | 0.01 | 2.022 | 0.043 |  |
|  | Cog slope →SI slope | -0.18 | -2.770 | 0.006 |  |
| Above senior high school level | |  |  |  |  |
|  | SI intercept →Cog intercept | -1.46 | -3.505 | < 0.001 | $\chi$^2^(100) = 146.08, *P* < 0.001; CFI = 0.91, TLI = 0.88, SRMR =0.048, RMSEA = 0.036 (0.023, 0.049) |
|  | SI intercept →Cog slope | 0.17 | 0.853 | 0.394 |  |
|  | SI slope →Cog slope | -0.84 | -0.943 | 0.346 |  |
|  | Cog intercept →SI intercept | -0.13 | -3.018 | 0.003 | $\chi$^2^(100) = 228.99, *P* < 0.001; CFI = 0.75, TLI = 0.67, SRMR =0.059, RMSEA = 0.061 (0.051, 0.071) |
|  | Cog intercept →SI slope | 0.02 | 0.675 | 0.500 |  |
|  | Cog slope →SI slope | 0.13 | 0.636 | 0.525 |  |
| *^a^* Adjusted for age, gender, smoking, alcohol use, number of non-communicable diseases, activities of daily living, and depression symptoms. | | | | | |

## eTable 5. Coefficients for structural models by health burden

| **Conditional model *^a^*** | **Parameters** | **Coefficients** | **Z value** | ***P* value** | **Goodness-of-fit indices** |
| --- | --- | --- | --- | --- | --- |
| None (no NCD) |  |  |  |  |  |
|  | SI intercept →Cog intercept | -1.40 | -9.718 | < 0.001 | $\chi$^2^(100) = 447.24, *P* < 0.001; CFI = 0.96, TLI = 0.95, SRMR =0.030, RMSEA = 0.034 (0.031, 0.037) |
|  | SI intercept →Cog slope | 0.24 | 3.868 | < 0.001 |  |
|  | SI slope →Cog slope | -2.08 | -3.537 | < 0.001 |  |
|  | Cog intercept →SI intercept | -0.07 | -8.413 | < 0.001 | $\chi$^2^(100) = 883.90, *P* < 0.001; CFI = 0.92, TLI = 0.90, SRMR =0.039, RMSEA = 0.051 (0.048, 0.054) |
|  | Cog intercept →SI slope | 0.01 | 0.493 | 0.622 |  |
|  | Cog slope →SI slope | -0.16 | -2.359 | 0.018 |  |
| Mild (1 or 2 types of NCDs) | |  |  |  |  |
|  | SI intercept →Cog intercept | -1.36 | -12.284 | < 0.001 | $\chi$^2^(100) = 699.95, *P* < 0.001; CFI = 0.96, TLI = 0.95, SRMR =0.034, RMSEA = 0.036 (0.033, 0.038) |
|  | SI intercept →Cog slope | 0.14 | 2.919 | 0.004 |  |
|  | SI slope →Cog slope | -2.56 | -3.193 | 0.001 |  |
|  | Cog intercept →SI intercept | -0.08 | -11.315 | < 0.001 | $\chi$^2^(100) = 1557.34, *P* < 0.001; CFI = 0.91, TLI = 0.88, SRMR =0.043, RMSEA = 0.056 (0.053, 0.058) |
|  | Cog intercept →SI slope | 0.01 | 2.274 | 0.023 |  |
|  | Cog slope →SI slope | -0.29 | -2.580 | 0.010 |  |
| Severe (more than three types of NCDs) | |  |  |  |  |
|  | SI intercept →Cog intercept | -1.35 | -7.386 | < 0.001 | $\chi$^2^(100) = 291.08, *P* < 0.001; CFI = 0.97, TLI = 0.96, SRMR =0.037, RMSEA = 0.034 (0.029, 0.038) |
|  | SI intercept →Cog slope | 0.13 | 1.602 | 0.109 |  |
|  | SI slope →Cog slope | -1.89 | -1.193 | 0.233 |  |
|  | Cog intercept →SI intercept | -0.07 | -6.446 | < 0.001 | $\chi$^2^(100) = 623.90, *P* < 0.001; CFI = 0.91, TLI = 0.88, SRMR =0.049, RMSEA = 0.056 (0.052, 0.060) |
|  | Cog intercept →SI slope | 0.01 | 1.110 | 0.267 |  |
|  | Cog slope →SI slope | -0.10 | -0.897 | 0.369 |  |
| *^a^* Adjusted for age, gender, education level, smoking, alcohol use, activities of daily living, and depression symptoms. | | | | | |
